# *Francisella* spp. as an overlooked cause of acute undifferentiated febrile illness in Colombia? Unexpected evidence from febrile patients negative for other common and neglected etiologies in Villeta municipality

**DOI:** 10.1186/s41182-025-00883-6

**Published:** 2026-01-03

**Authors:** Carlos Ramiro Silva-Ramos, Maria Camila Sierra-González, Miguel Esteban Chacón Gómez, Peter C. Melby, Patricia V. Aguilar, Miguel M. Cabada, Marylin Hidalgo

**Affiliations:** 1 Grupo de Enfermedades Infecciosas, Departamento de Microbiología, Facultad de Ciencias, Pontificia Universidad Javeriana, Carrera 7 No. 43 – 82, Bogotá, D.C. Colombia; 2 Department of Pathology, University of Texas Medical Branch, Galveston, TX USA; 3 Centro de Secueciación GenCore, Universidad de Los Andes, Bogotá, Colombia; 4 Division of Infectious Diseases, Department of Internal Medicine, University of Texas Medical Branch, Galveston, TX USA; 5 Center for Tropical Diseases, University of Texas Medical Branch, Galveston, TX USA

**Keywords:** Francisella, Acute undifferentiated febrile illness, 16s rRNA gene sequencing, Barcoding, Emerging infections, Zoonotic bacteria, Neglected pathogens, Colombia

## Abstract

**Background:**

Acute undifferentiated febrile illness (AUFI) represents a major health challenge in tropical regions due to its wide range of etiologies. In Villeta, Colombia, previous studies investigated common causes such as malaria, arboviral diseases, leptospirosis and rickettsiosis, as well as several neglected bacterial agents. However, some patients remained without an identified etiology, underscoring the need for broader approaches to uncover other potential causes. Therefore, the aim of the present study was to investigate into other potential bacterial causes of AUFI through advanced molecular strategies utilizing 16S rRNA sequencing.

**Methods:**

The study analyzed AUFI patient samples previously screened for fourteen pathogens. The V3–V9 hypervariable region of the 16S rRNA gene was amplified from whole-blood DNA of unresolved cases and sequenced using the Oxford Nanopore GridION platform. Reads were filtered, quality-checked, and taxonomically classified using the SILVA database.

**Results:**

Eight samples from individuals without evidence of infection or recent exposure to previously screened pathogens were selected for 16S rRNA sequencing. DNA quality and integrity were confirmed, and enrichment produced high-quality amplicons for all samples. Sequencing generated high-quality reads overwhelmingly dominated by *Francisella*, representing over 93% of classified reads, followed by *Coxiella* and *Arcobacter*.

**Conclusions:**

This study provides the first molecular evidence of *Francisella* in whole-blood from febrile patients in Colombia. Findings highlight its potential role in AUFI, demonstrate the value of 16S rRNA barcoding, and underscore the need for expanded surveillance of highly neglected bacterial taxa.

**Supplementary Information:**

The online version contains supplementary material available at 10.1186/s41182-025-00883-6.

## Introduction

Acute undifferentiated febrile illness (AUFI) is the most frequent cause of medical attention in tropical and subtropical regions worldwide, representing a major clinical and public health challenge [1, 2]. Patients typically experience fever along with non-specific symptoms such as headache, myalgia, and general malaise [1, 3]. The early clinical presentation of AUFI is unspecific, hindering clinical diagnosis, prognosis assessment and biosafety decisions, particularly in resource constrained areas, as the disease can range from mild and self-limiting forms to severe or even fatal outcomes. In this settings, diagnosis may remain a challenge due to limited capacity for laboratory confirmation [1, 2].

AUFI has the potential to evolve into a life-threatening illness. Thus, the early identification of its specific etiology is crucial to guide appropriate treatment or timely referral to higher-level health centers, to prevent fatal outcomes or chronic complications [1, 4]. The etiology of AUFI is diverse, involving both globally distributed and geographically restricted tropical pathogens [1]. Clinical algorithms to assist healthcare personnel making accurate clinical diagnosis without laboratory confirmation remains a significant hurdle [3].

Malaria remains a significant cause of AUFI in Latin America and the Caribbean with approximately 120 million people at risk [5]. Nevertheless, recent large outbreaks of arboviral infections in the region have shifted the focus of health authorities. Dengue virus (DENV) is now considered the most frequent cause of febrile illness in tropical regions, followed by Chikungunya (CHIKV) and Zika (ZIKV) viruses, which have gained considerable relevance since their emergence [6, 7]. The predominance of these arboviruses has created an “umbrella effect,” leading to other endemic etiologies being overlooked or inadequately considered [8, 9]. This includes leptospirosis and rickettsioses, which are global zoonoses with increasing importance in rural areas of Latin America [10, 11]. Moreover, novel and emerging pathogens have expanded the etiological spectrum of AUFI. For instance, the emergence of Severe Acute Respiratory Syndrome Coronavirus 2 (SARS-CoV-2) has added complexity to AUFI diagnosis in tropical regions due to its global distribution and overlapping clinical presentation [12, 13]. The recognition of scrub typhus in southern Chile, with potential for a wider distribution [14, 15], and the resurgence of Oropouche virus (OROV) in the Amazon basin [16, 17], further complicates specific diagnosis. Moreover, other arboviruses endemic in several Latin American countries such as Mayaro, Guaroa, and Madariaga viruses remain insufficiently monitored [18–21].

Conventional diagnostic methods like microbiology, serology and molecular tests often fall short in pinpointing the exact cause of AUFI; therefore, strategies such as next-generation sequencing (NGS), specifically metagenomics and metabarcoding sequencing, have emerged as highly valuable tool for detecting this broad range of pathogens, especially when routine methods fail [22–24]. Beyond the identification, these advance tools offer crucial preliminary insights into local epidemiology. This is particularly important in low and middle-income countries where economical constrains often limit access to conventional diagnostics. By understanding the local pathogen landscape better, more targeted diagnostic algorithms can then be developed [25, 26].

Colombia’s incredible biodiversity, coupled with its tropical and isothermal climate across varying altitutes and temperatures, create an ideal environment for a variety of pathogens linked to AUFI [27, 28]. While the Colombian national surveillance system monitors some AUFI-related etiologies such as dengue, malaria and leptospirosis, regional studies have consistently shown that many other pathogens are actively circulating but are not formally reported [29]. Therefore, the full spectrum of AUFI etiologies remains incompletely characterized, and it is likely that additional, unrecognized pathogens are contributing to the disease burden.

In the municipality of Villeta, two studies have attempted to characterize the etiology of AUFI [30, 31]. In the first study, even after targeting five different pathogens, nearly 28% (29/104) of febrile patients remained without a specific diagnosis [30]. A more recent study, screening for ten different etiologies, still left a significant 44.6% (25/56) of patients without a definitive cause for their fever [31]. Even with subsequent analyses using more sensitive methods and targeting additional neglected pathogens, a notable proportion of patients continued to lack a recognized cause [32, 33] (Silva-Ramos et al. manuscript in preparation). This persistent diagnostic gap highlights the critical need for alternative approaches. Therefore, the present study aims to investigate deeper into other potential bacterial causes of AUFI through advanced molecular diagnostics using ribosomal gene sequencing, to broaden the understanding of the bacterial agents associated with AUFI in this region.

## Materials and methods

### Study area

**Fig. 1:**
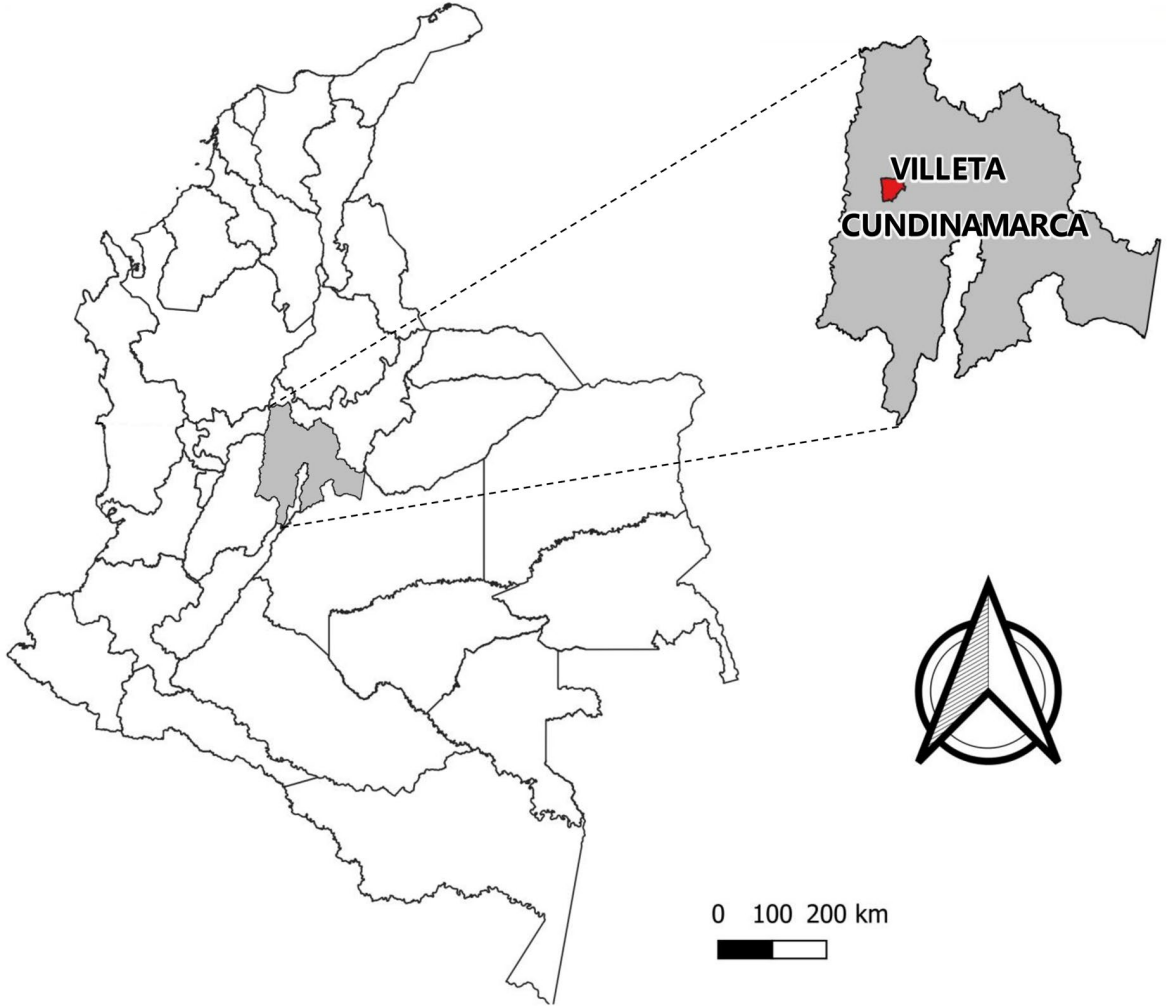
Map showing the location of Villeta municipality (highlighted in red) within Cundinamarca department (highlighted in gray)

Villeta (5° 00′ 46″ N, 74° 28′ 23″ W) (Fig. 1), is a municipality located at an elevation of 850 m above sea level (MASL), in the Gualivá Province, Cundinamarca Department, 84 km from Bogotá D.C. It covers 140 km^2^ distributed in 22 villages. The annual mean temperature is 26 °C, with a relative humidity ranging from 80 to 97%. Eco-tourism and agriculture are the main economic activities, with sugarcane cultivation and “panela” production being the predominant economic activities in rural areas (www.villeta-cundinamarca.gov.co).

### Samples

Samples were collected as part of a larger multisite study on AUFI conducted in six regions across four different countries, following a common protocol and data collection system [34]. Villeta was selected due to its better accessibility for conducting the study. Briefly, between September and December 2021, active surveillance for AUFI was carried out at “Salazar de Villeta” Hospital. Patients were evaluated in the emergency department for febrile illness with no clear focus of infection. Inclusion criteria were age > 2 years and documented fever > 37.8 °C with less than 14 days of duration, while exclusion criteria included patients < 2 years, those in whom an identifiable source of infection was evident (e.g., otitis media, purulent pharyngitis, urinary tract infection, among others), and individuals who did not agree to voluntarily participate in the study or did not sign the informed consent. A total of 56 patients were included in the study, all of them were residents of Villeta municipality. For each recruited patient, clinical, demographic, and epidemiological data were recorded, and acute whole-blood, acute serum and convalescent serum samples were obtained as part of a previous study [31]. These samples were stored at the “Special Bacteriology Laboratory (Laboratorio de Bacteriología Especial)” of the “Pontifical Xavierian University (Pontificia Universidad Javeriana)” in Bogotá D.C., Colombia.

The study protocol, informed consent and assent were approved by the ethics committee of the Pontificia Universidad Javeriana. Written informed consent was obtained from all patients, and from parents or legal guardians in the case of minor-age patients. Some participants provided consent for future use of their samples in other studies. All data were deidentified by assigning a unique numeric code to each patient. All procedures adhered to the resolution 8430 from 1993 of the Ministry of Health of Colombia and the declaration of Helsinki for ethical principles in human research.

### Sample selection

A broad screening for multiple infectious agents associated with AUFI was previously performed among the 56 recruited febrile patients, including fourteen infectious agents: SARS-CoV-2, *Plasmodium*, Dengue virus, Chikungunya virus, Yellow fever virus, Oropouche virus, Mayaro virus, Venezuelan equine encephalitis virus, *Leptospira*, *Rickettsia* [31, 32], *Orientia* [33], *Bartonella*, *Borrelia*, and *Coxiella burnetii* (Silva-Ramos et al., manuscript in preparation). Of the 56 febrile patients, 26 showed no evidence of current infection or recent exposure to any of these fourteen screened agents. Among them, fourteen patients authorized the use of their samples in future studies, and based on sample availability, eight whole-blood DNA samples were selected for massive sequencing targeting the 16S rRNA ribosomal gene (barcoding), to explore the presence of additional bacterial taxa not included in the whole screening performed.

### DNA quality assessment and quantification

The integrity of the extracted DNA was evaluated by electrophoresis on 1% agarose gels, which allowed the detection of high-molecular-weight fragments and the exclusion of degraded products. Samples with adequate DNA integrity were further assessed for concentration and purity. DNA concentration was measured using a Qubit™ 4.0 Fluorometer (Thermo Fisher Scientific, Waltham, MA, USA) with the Qubit™ dsDNA HS Assay Kit, following the manufacturer’s instructions and using 1 μL of each sample for quantification. DNA purity was assessed with a NanoDrop 2000 spectrophotometer (Thermo Fisher Scientific, Waltham, MA, USA) by calculating the A260/280 and A260/230 absorbance ratios. Results were classified according to the nanopore sequencing quality criteria defined by the “DNA Sequencing Core Facility (Core de Secuenciación GenCore)” from the “University of the Andes (Universidad de los Andes)” into four levels: level A or adequate concentration without degradation or contaminants; level B or slightly degraded and/or low DNA quantity; level C or presence of detectable contaminants and/or minimal DNA quantity; and level D or evident degradation and/or insufficient DNA concentration. Only samples classified as level A, B or C were selected for 16S rRNA gene amplification.

### Amplification of the 16S rRNA gene

Amplification of the full 16S rRNA gene, (~ 1500-bp) was performed using the universal primers 27-F (AGAGTTTGATCMTGGCTCAG) and 1492-R (TACGGYTACCTTGTTACGACTT) [35]. For samples without consistent products, amplification of the V3–V9 hypervariable region (~ 1160-bp) was performed using primers 337F (GACTCCTACGGGAGGCWGCAG) and 1492R (TACGGYTACCTTGTTACGACTT) [36], considering the higher taxonomic resolution provided by this region.

Samples yielding the expected amplicon but showing nonspecific banding patterns were additionally purified using AMPure XP magnetic beads (Beckman Coulter Life Sciences, Barcelona, Spain) at a 0.4 × ratio. Additionally, samples showing faint amplification bands were submitted to an extra enrichment step to maximize the performance of subsequent procedures.

### Library preparation and sequencing

Library preparation was performed using the Native Barcoding Kit 24 V14 (SQK-NBD114.24) (Oxford Nanopore Technologies, Oxford, UK), following the manufacturer’s recommendations. This kit enables multiplexing of multiple samples in a single sequencing run through the incorporation of molecular barcodes, allowing individual identification during de-multiplexing. Each processed sample was assigned a unique barcode, followed by adapter ligation and fragment purification. All available genetic material was used to maximize sequencing output.

Sequencing was performed using the GridION platform (Oxford Nanopore Technologies, Oxford, UK), which provides real-time long-read sequencing and supports multiple simultaneous runs. The MinION/GridION R10.4.1 flow cell (Shirley, NY, USA) was used, ensuring the minimum number of active nanopores required for high-quality data and sufficient coverage. These flow cells are optimized for high-fidelity sequencing, particularly in GC-rich regions or sequences with complex secondary structures.

Sequencing was conducted with the MinKNOW software (Oxford Nanopore Technologies, Oxford, UK), applying a minimum read length filter of ≥ 200-bp and a quality threshold (Q-score) ≥ 9. Base-calling was performed with the Dorado v7.9.8 software (Oxford Nanopore Technologies, Oxford, UK) using the high-accuracy model. Raw output files (.pod5) were demultiplexed according to assigned barcodes, and obtained reads were then classified into two lists, fastq_pass (Q-score ≥ 9) and fastq_fail (below threshold). Only fastq_pass reads were included in the following bioinformatic analyses.

### Quality control and filtering

All files obtained after sequencing were verified for correct organization by barcode within the input directory, including fastq read files and the general sequencing report. Reads from each sample were merged into a single fastq file per barcode and stored in the input_merged directory. Internal validation ensured that the number of merged reads matched the original counts, avoiding data loss during merging.

Read quality was assessed with the NanoPlot software [37], which generated several metrics per barcode which included total reads, average length, quality score distribution, and N50 values. Using Porechop, filtering criteria were then applied to exclude low-quality and non-representative reads, which included those reads with a Q-score < 12, sequences that fell outside the expected amplicon length range between 1100 and 1450-bp, and reads that contained adapters or repetitive sequences. Only the reads that met these criteria were retained for taxonomic assignment analysis.

### Taxonomic assignment

Taxonomic classification of filtered sequencing reads was carried out employing Emu, a specialized bioinformatics tool specifically designed and optimized for the characterization of microbial communities and barcoding analysis of long-read nanopore sequences [38]. The sequencing reads corresponding to each individual barcode were subsequently compared against the SILVA 138.2 SSU NR99 database [39], which is a curated and widely recognized 16S rRNA sequencing repository that has been extensively used in studies of bacterial and archaeal diversity ensuring a high level of quality, consistency and reliability in the taxonomic classification process [40].

To reduce the impact of sporadic reads potential sequencing errors or artifactual assignments, a minimum relative abundance threshold of 0.1% was applied. Those taxonomic assigntments below this established threshold were excluded from further analyses to avoid spurious results and to prevent the potential overinterpretation of minor and non-representative signals obtained within the datase.

The final taxonomic resolution of the data was initially restricted to the bacterial genus level, to enhance both robustness and the overall accuracy of the taxonomic classification obtained. Additionally, those bacterial genera that were identified as particularly relevant for the etiology of AUFI, or those containing medically relevant pathogens were further examined to a more detailed analysis at the species level to explore the potential presence of specific infectious agents of clinical and epidemiological importance.

## Results

### DNA quality and integrity

Evaluation of DNA integrity by 1% agarose gel electrophoresis confirmed the presence of high-molecular-weight genomic DNA in all eight processed samples, with no visible evidence of degradation. Fluorometric quantification using the Qubit™ dsDNA HS Assay Kit showed variable DNA concentrations ranging from 0.22 to 4.12 ng/µL. Spectrophotometric purity assessment revealed A260/280 ratios around 1.88 and A260/230 ratios between 0.31 and 1.14. Based on the predefined quality criteria considering DNA integrity, purity and concentration, each sample was classified as level C quality. All eight samples met the minimum requirements for downstream amplification and were therefore included in subsequent steps. Individual information on DNA integrity, purity, concentration and final quality classification are provided in provided in Supplementary Table 1.

### 16s rRNA amplification

**Fig. 2:**
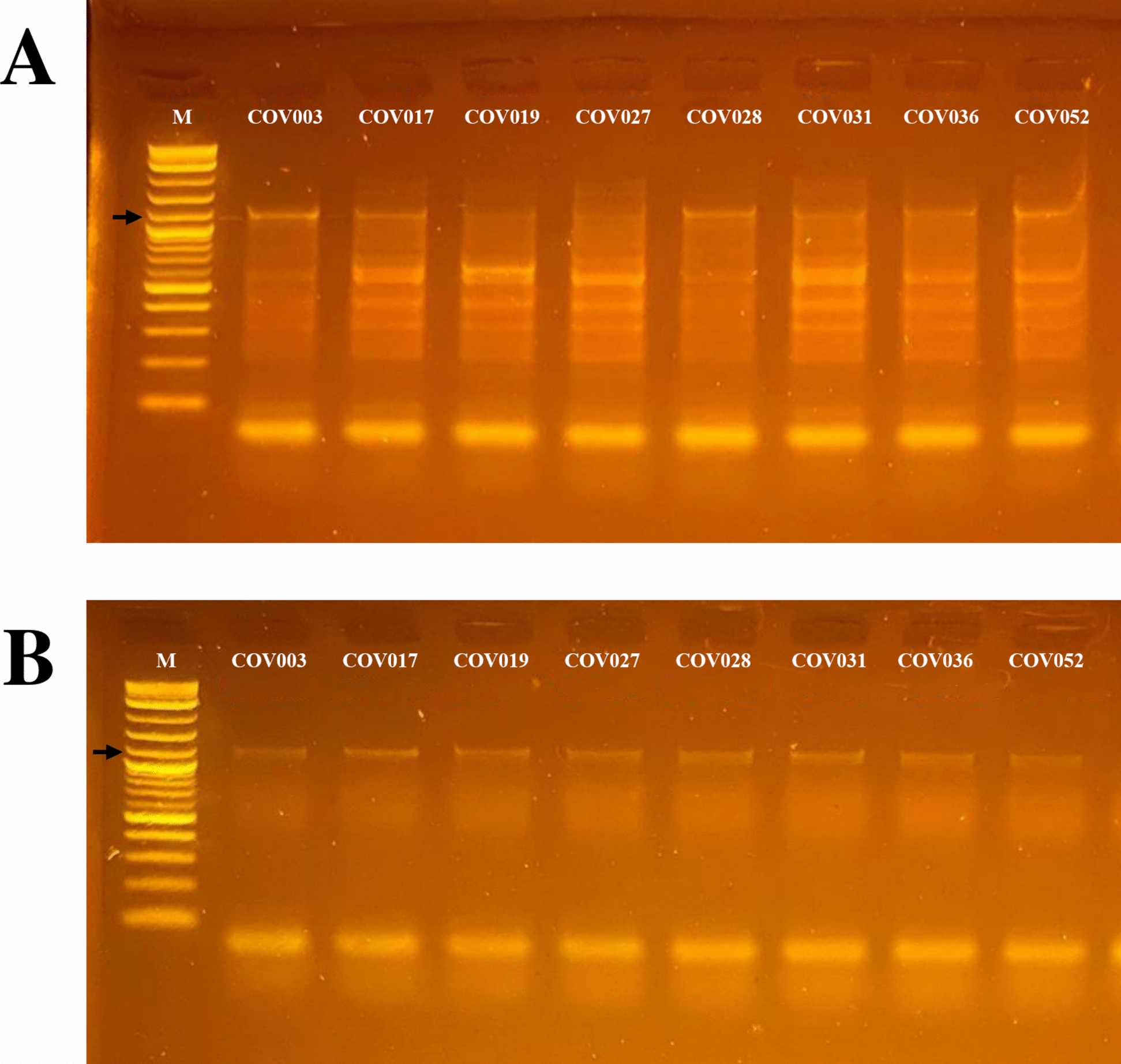
Agarose gel electrophoresis of PCR products corresponding to the bacterial 16S rRNA V3–V9 region with an expected amplicon size of ~ 1160 bp. **A** Amplicons before purification and enrichment. **B** Amplicons after purification and enrichment. Lane M: molecular marker 1 kb Plus DNA Ladder (New England Biolabs, Ipswich, MA, USA), with the 1100-bp band indicated by a black arrow. Lanes 1–8: amplified samples

Of the eight samples that passed the DNA quality and integrity control, amplification of the full-length 16S rRNA gene was not successful in any of them. In contrast, amplification of the partial V3–V9 region was successfully achieved in all eight analyzed samples. Some obtained PCR products showed variable banding patterns, with the expected ~ 1160-bp fragment present in all eight samples but accompanied by additional nonspecific bands in some samples, and with the expected fragment appearing faint in others (Fig. 2A, ampllicons before enrichment and purification). These samples were subjected to an additional enrichment and purification process, and as a result, a single, consistent, high-quality amplicon corresponding to the expected target fragment was obtained in all eight samples, making them suitable for subsequent library preparation (Fig. 2B, amplicons after enrichment an purification).

### Amplicon reads from sequence data

A total of 11 µL per sample was used for library construction. Each sample was assigned a unique barcode, allowing individual identification of each processed sample within the sequencing. The complete sequencing and base-calling process lasted a total of 3 days, 4 min and 2 s, with no record of technical incidents. A total of 6.29 million reads, equivalent to 7.43 billion bases of raw sequence data, was generated. Of these, 5.51 million reads or 6.13 billion bases of sequence data, passed the minimum quality threshold established. The estimated N50 was ~ 1290-bp. All these data reflect adequate amplification integrity and consistent platform performance. General sequencing parameters and quality metrics are summarized in Table 1.

**Table 1:** Summary of sequencing run performance and quality metrics

Parameter	Obtained value
FlowCell ID	FBA99703
Sequencing run time	3d 0 h 4 m 3 s
Total reads generated	2.24 M
fastq_pass	1.54 M
fastq_fail	696.9 K
Total estimated bases generated	2.58 Gb
High-quality bases	1.81 Gb
Low-quality bases	0.76 Gb
Estimated N50	1.29 Kb

### Data quality

The number of pre-merge reads and bases matched the number of post-merge data in all cases, confirming correct data consolidation for each barcode. After applying quality and length filters, sufficient data volume remained for further taxonomic assignment analyses, with an overall read retention rates ranged between 53 and 71.8%. Considering the information content, filtered bases ranged between 11.8 and 369.9 million base pairs. Dataset obtained after filtering showed consistency in read length with the expected size of the amplified fragment, around 1173-bp, with the mean post-filtering quality score stable across samples. More information can be observed in Supplementary Table 2.

### Taxonomic assignment

**Fig. 3:**
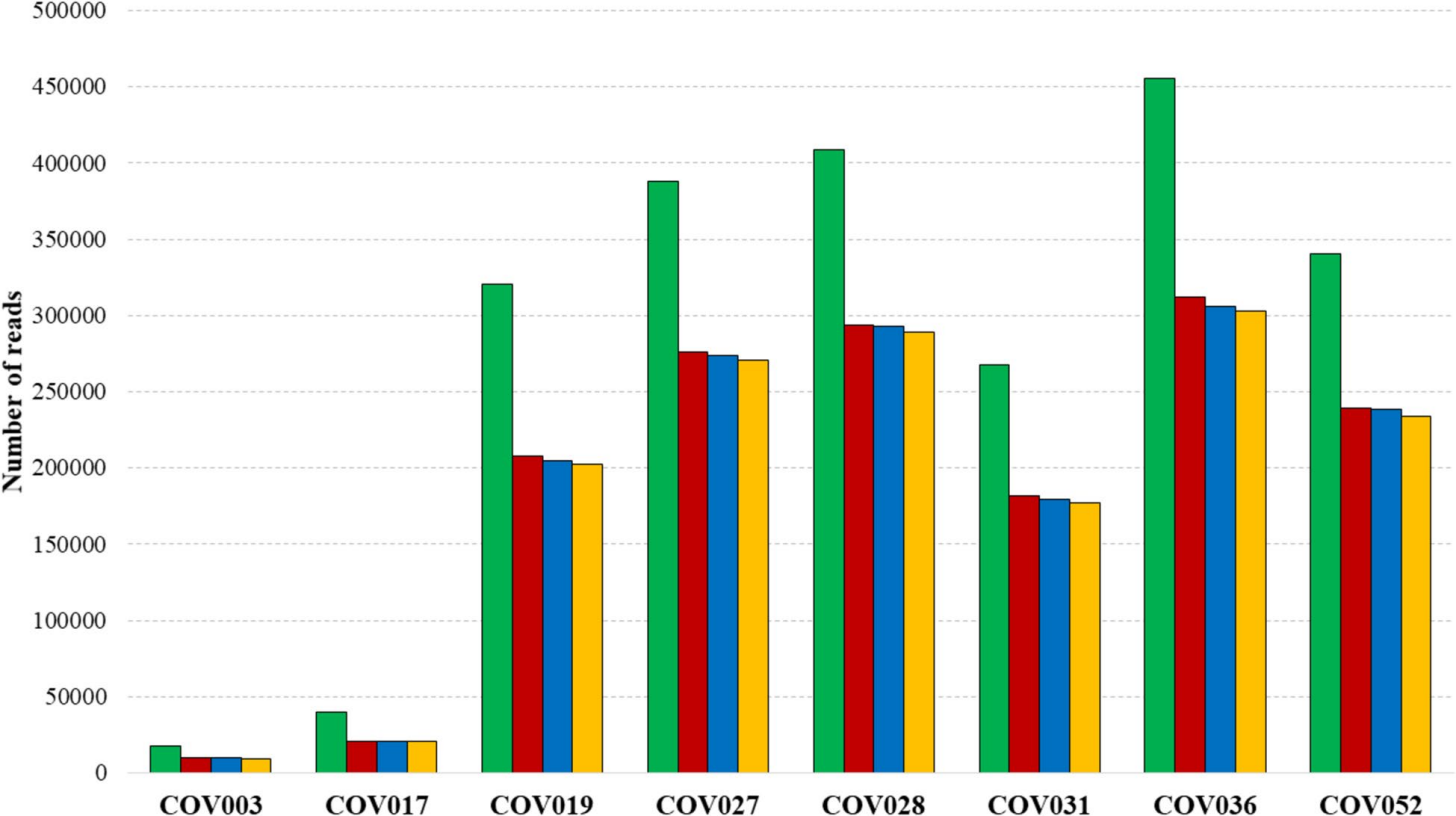
Bar charts showing the number of total reads obtained (shown in green), filtered reads (shown in red), mapped reads (shown in blue), and classified reads (shown in yellow) for each sample analyzed. Exact numerical values for each sample are provided in Supplementary Tables S2 and S3

A total of eight samples were included in the 16S rRNA barcoding analysis. More than 97% of the filtered reads were taxonomically classified accordingly and successfully matched at least one reference sequence from the database (Fig. 3).

**Fig. 4:**
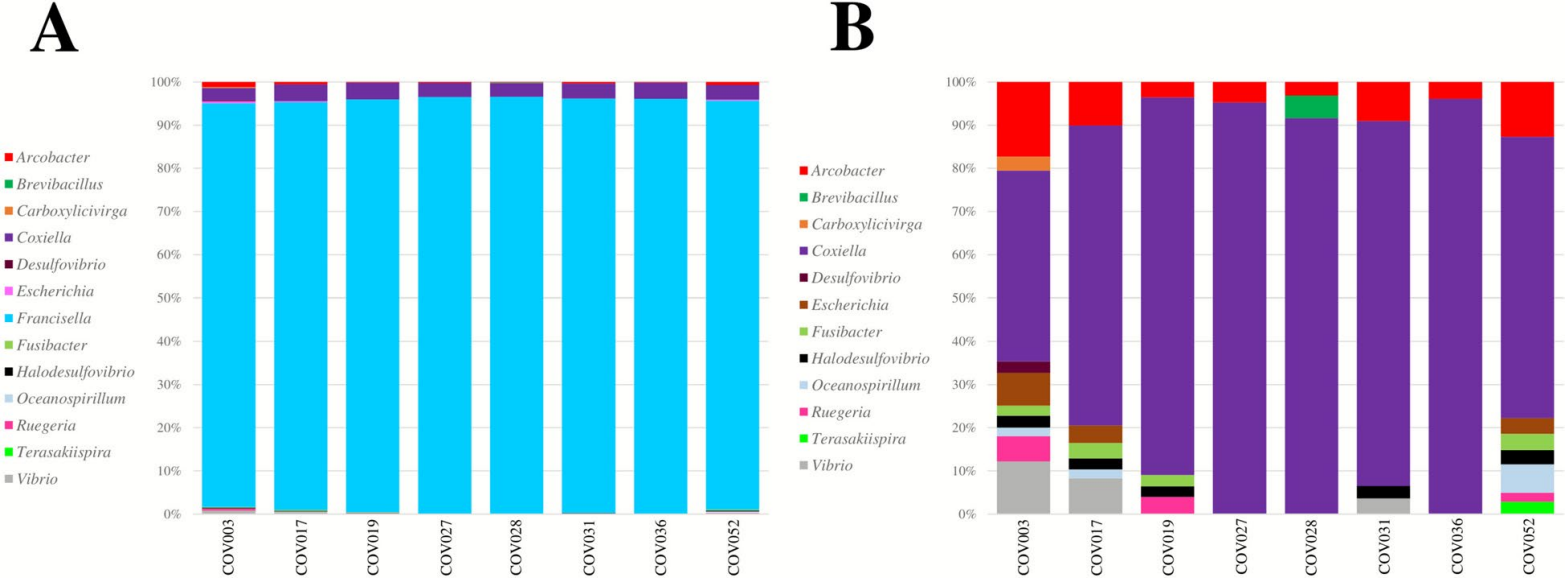
Distribution patterns and taxonomic composition of bacterial genera detected in each febrile patient sample based on 16s rRNA barcoding after excluding fourteen common and neglected pathogens through prior screening, as determined by 16S rRNA barcoding sequencing. **A** Including all assigned genera. **B** Excluding *Francisella* to better visualize less abundant taxa. In both panels, each bacterial genus is represented by a different color

Filtered reads were assigned to thirteen bacterial genera (Fig. 4A). The number of bacterial genera assigned ranged between three to ten per sample. Across all samples, the bacterial assignment was consistently dominated by the genus *Francisella*, which was assigned in 100% (8/8) of all processed samples, representing over 93% of classified reads in every case (Fig. 4A). Although at much lower relative abundance, *Coxiella* was classified as the second most frequent bacterial genus, followed by *Arcobacter*, both appearing consistently across all samples (Fig. 4B). Taxonomic assignment also revealed, at lower relative abundances in some samples, the presence of two medically relevant bacterial genera, *Escherichia* and *Vibrio* (Fig. 4B). An overview of the mapping and taxonomic classification metrics per sample can be observed in Supplementary Table 3.

Despite the limited resolution of 16S barcoding for species-level discrimination, an exploratory assessment of bacterial diversity was performed to provide a preliminary overview of the potential species present in the samples. Among the eight febrile patients analyzed, 23 distinct species-level taxa were detected. Some reads were assigned to five recognized pathogenic species: *Escherichia coli*, *Francisella hispaniensis*, *Vibrio alginolyticus*, *Vibrio harveyi* and *Vibrio parahaemolyticus*. When focusing on the most abundant taxa, the majority of reads were classified as *Francisella* and *Coxiella* endosymbionts associated with *Amblyomma maculatum*, with relative abundances of > 92% and 2.7%, respectively, in all samples, representing the majority of reads in the dataset obtained. A summary of the demographic, epidemiological, and clinical characteristics of the eight patients included in this analysis is presented in Table 2.

**Table 2:** Summary of febrile patient demographics, epidemiological data and clinical features

Sample ID	Age group	Gender	Origin	Occupation	Recruitment month	Exposure history	Fever characteristics	Other clinical manifestations	Outcome
COV003	Adolescent (13)	Female	Urban	Student	September	Mosquitoes, cats	3-day fever, sudden onset, intermittent, associated with total inactivity	Chills, malaise, fatigue, nausea, abdominal pain, myalgia, headache	Resolved
COV017	Young adult (20)	Male	Urban	Health worker	November	Mosquitoes, dogs	2-day fever, sudden onset, intermittent, associated with total inactivity	Chills, malaise, anorexia, sweats, fatigue, prostration, retro-ocular pain, cough, abdominal pain, diarrhea, coluria, myalgia, headache	Resolved
COV019	Young adult (18)	Male	Urban	Construction worker	November	Mosquitoes, rodents, birds, dogs, cats	3-day fever, gradual onset, intermittent, associated with total inactivity	Malaise, anorexia, sweats, fatigue, prostration, retro-ocular pain, hemoptysis, dyspnea, cough, chest pain, nausea, vomiting, myalgia, arthralgia, headache	Resolved
COV027	Young adult (24)	Male	Urban	Administrative worker	December	Mosquitoes, dogs	2-day fever, gradual onset, continuous, associated with partial inactivity	Chills, malaise, anorexia, sweats, fatigue, prostration, nausea, papular rash, headache	Resolved
COV028	Old adult (45)	Male	Urban	Transportation	December	Mosquitoes, dogs	2-day fever, sudden onset, intermittent, associated with partial inactivity	Chills, malaise, fatigue, nausea, myalgia	Resolved
COV031	Young adult (25)	Male	Urban	Student	December	Mosquitoes, fleas, dogs, cats	2-day fever, sudden onset, intermittent, associated with partial inactivity	Chills, malaise, fatigue	Resolved
COV036	Adolescent (13)	Female	Urban	Student	December	Mosquitoes, dogs	1-day fever, gradual onset, intermittent, associated with normal inactivity	Malaise, sweats, fatigue, nausea, abdominal pain, diarrhea, vomiting, agitation	Resolved
COV052	Young adult (19)	Female	Urban	Student	December	Mosquitoes, dogs	3-day fever, sudden onset, continuous, associated with total inactivity	Chills, malaise, anorexia, sweats, fatigue, prostration, conjunctivitis, cough, chest pain, abdominal pain, diarrhea, coluria, macular rash, myalgia, arthralgia, headache, petechiae	Resolved

## Discussion

AUFI represents a major challenge in tropical and subtropical regions globally, largely due to the wide range of pathogens involved [41–43]. In the municipality of Villeta, previous studies have focused primarily on rickettsial infections [44, 45]; however, a substantial knowledge gap remains regarding the contribution of other bacterial taxa in the local epidemiology of AUFI. In the present study, we analyzed previously undiagnosed AUFI samples from Villeta and identified additional bacterial taxa using a 16S rRNA barcoding approach, revealing the presence of several bacterial genera, some of which may be epidemiologically relevant in this setting.

Surprisingly, *Francisella* spp. overwhelmingly dominated the bacterial taxa in the processed samples, accounting for over 93% of the classified reads. *Francisella* is a genus composed of at least 20 species, many of which are typically associated with illness in fish [46]. However, at least seven species are known to cause disease in humans. These include *Francisella hispaniensis*, *Francisella philomiragia*, *Francisella salimarina*, and most important, the *Francisella tularensis* complex (*F. tularensis* subsp. *holarctica*, *F. tularensis* subsp. *mediasiatica*, *F. tularensis* subsp. *novicida* and *F. tularensis* subsp. *tularensis*), which causes tularemia [47–49]. *F. tularensis* is a known cause of zoonotic AUFI in various countries across the Northern Hemisphere, including parts of the Americas, Europe and Asia [50, 51]. Interestingly, cases have also been reported in some African countries, primarily in the northern region [52, 53]. There is even a report from Kenya, further south, where serological evidence of *F. tularensis* was found in 3.7% (27/730) of febrile patients [54]. Furthermore, confirmed tularemia cases have also been reported in Australia, linked to contact with small marsupials [55, 56]. These findings support the idea that *F. tularensis* might have a much broader distribution, extending into the Southern Hemisphere as well.

In South America, our understanding of medically relevant *Francisella* species is limited. To date, only two studies from Uruguay have documented *Francisella* infections: one detailed a clinical case of septic arthritis caused by a unique *F. philomiragia* strain [57], and the other provided pathological and immunohistochemical evidence of infection in aborted sheep fetuses [58]. In Colombia, the presence of *Francisella* endosymbionts has been documented in two different tick groups, *Dermacentor nitens* collected from horses [59] and *Amblyomma* spp. sampled from wildlife [60]. In our study, most reads obtained were classified as *Francisella* endosymbionts of *Amblyomma maculatum.* This finding is significant because it suggests these endosymbionts might retain, or even reacquire, their infectious potential, and thus, its role in human or animal disease cannot be ruled out [61]. This new data, combined with previous evidence of *Francisella* serological reactivity in nearly 40% (107/271) of febrile patients in the Magdalena Medio region [62], strongly suggests that not only are non-pathogenic *Francisella* endosymbionts present, but a strain with underestimated clinical relevance could be actively circulating in Colombia. The detection of *Francisella* sequences in febrile patients from Villeta municipality marks the first molecular evidence of this bacterial genus in vertebrate hosts in Colombia. This strongly suggests the presence of at least one species capable of infecting humans in the country. Given our limited knowledge of *Francisella* in Colombia, the possibility that these sequences represent a novel undescribed species of public health importance cannot be ruled out. This study opens a new avenue for future research to characterize this bacterial genus and clarify its role in the etiology of zoonotic AUFI in the country.

Beyond *Francisella*, we consistently found sequences classified as other medically relevant bacterial genera, specifically *Coxiella* and *Arcobacter,* though in lower proportions across all processed samples. The *Coxiella* sequences obtained were classified as endosymbionts of *Amblyomma americanum*, which has been identified as an important lineage that provides essential vitamins to their tick hosts but without evidence they can infect vertebrates [63, 64]. This aligns with previous studies performed in Villeta [30] (Silva-Ramos et al. manuscript in preparation), which found no molecular or serological evidence of *Coxiella burnetii* infection. Therefore, it is likely these sequences may correspond to *Coxiella* endosymbionts. Their detection in the blood of febrile patients probably indicates a transient transfer of bacterial DNA from feeding ticks, rather than active infection, serving as an indirect marker of tick exposure. On the other hand, *Arcobacter* reads, while not directly linked to AUFI, warrants further attention. This genus is gaining recognition as an emerging human pathogen, primarily associated with gastrointestinal illness, but also capable of causing more severe conditions like bacteremia and endocarditis [65, 66]. Its consistent detection in all processed samples raises questions about its potential role in systemic infections in Colombia, and warrants further investigation.

Unfortunately, all available DNA from these patients was used during the present analyses and no additional material remained to perform targeted molecular assays for independent confirmation. Therefore, the results obtained through 16S rRNA barcoding should be interpreted as preliminary and exploratory, and further confirmation using independent methods are required in future studies. Overall, these findings underscore the high potential of 16S rRNA barcoding as a complementary tool to expand our understanding of potential bacterial pathogens associated with AUFI. The evidence obtained opens up exciting new avenues for studying highly neglected bacterial taxa, especially *Francisella* spp., in Colombia. Future efforts should integrate metagenomics for higher-resolution identification. It would also be crucial to attempt bacterial culture and isolation to determine the exact species involved in human infections. Additionally, sero-epidemiological studies are needed to assess exposure levels in local human populations, and given *Francisella’s* zoonotic capacity, investigating potential animal reservoirs is essential. Expanding our sampling framework to include vectors and wildlife hosts would further strengthen our understanding of the significance of *Francisella* and other bacterial taxa in the epidemiology of AUFI.

## Conclusion

This study offers the first molecular evidence of *Francisella* spp. in whole blood samples from febrile patients in Colombia, which highlights the potential role of this bacterial genus as a cause of AUFI in the region. The consistent dominance of *Francisella* signals across all samples analyzed, combined with earlier serological findings from another part of the country, strongly suggests that both endosymbiotic and potentially pathogenic lineages capable of infecting humans might be circulating locally. Our results clearly demonstrate the value of 16S rRNA barcoding approach as a complementary tool to detect etiologies of AUFI. The detection of additional genera like *Coxiella* and *Arcobacter* reinforces the need to consider bacteria that are often neglected or underestimated in future studies. Altogether, these findings emphasize the importance of expanding surveillance efforts through metagenomics, bacterial isolation, and sero-epidemiological studies in humans, animals and vectors. This comprehensive approach will be crucial for determining the true clinical and ecological significance of these bacterial taxa signals detected in Colombia.

## Supplementary Information

**Additional file 1**. Table 1: DNA integrity, purity, quantification and quality classification parameters of 16S rRNA barcoding selected samples from febrile patients negative for other common and neglected AUFI-related pathogens.

**Additional file 2**. Table 2: General sequencing output parameters and quality control metrics obtained from 16S rRNA barcoding.

**Additional file 3**. Table 3: Mapping and taxonomic classification metrics obtained for each processed sample.

## Data Availability

The datasets used and/or analysed during the current study are available from the corresponding author on reasonable request.

## Abbreviations

A260/230: Absorbance ratio at 260 and 230 nm
A260/280: Absorbance ratio at 260 and 280 nm
AUFI: Acute undifferentiated febrile illness
bp: Base pairs
CHIKV: Chikungunya virus
DNA: Deoxyribonucleic acid
DANE: *Departamento Administrativo Nacional de Estadística*
DENV: Dengue virus
NGS: Next-generation sequencing
MASL: Meters above sea level
ng/µL: Nanograms per microliter
OROV: Oropouche virus
PCR: Polymerase chain reaction
Q-score: Quality score
RNA: Ribonucleic acid
rRNA: Ribosomal RNA
SARS-CoV-2: Severe Acute Respiratory Syndrome Coronavirus 2
V3–V9: Variable regions 3–9 of the 16S rRNA gene
ZIKV: Zika virus
