# Supplementary material for: Francisella spp. as an overlooked cause of acute undifferentiated febrile illness in Colombia? Unexpected evidence from febrile patients negative for other common and neglected etiologies in Villeta municipality
Source: Trop Med Health. 2026 Jan 3;54:16. doi: 10.1186/s41182-025-00883-6 (PMC12805694; doi:10.1186/s41182-025-00883-6)
Supplement: Supplementary file 1 — Additional file 1. Table 1: DNA integrity, purity, quantification and quality classification parameters of 16S rRNA barcoding selected samples from febrile patients negative for other common and neglected AUFI-related pathogens. [file 41182_2025_883_MOESM1_ESM.docx]

**Supplementary Table 1.** DNA integrity, purity, quantification and quality classification parameters of 16S rRNA barcoding selected samples from febrile patients negative for other common and neglected AUFI-related pathogens.

| **Sample ID** | **DNA integrity** | **A260/280 ratio** | **A260/230 ratio** | **DNA concentration (ng/µL)** | **Quality classification** |
| --- | --- | --- | --- | --- | --- |
| COV003 | Yes | 1.87 | 0.45 | 0.22 | C |
| COV017 | Yes | 1.96 | 0.65 | 0.60 | C |
| COV019 | Yes | 1.85 | 0.78 | 2.34 | C |
| COV027 | Yes | 1.90 | 0.80 | 3.24 | C |
| COV028 | Yes | 1.76 | 0.31 | 3.66 | C |
| COV031 | Yes | 1.83 | 0.72 | 4.12 | C |
| COV036 | Yes | 1.90 | 1.14 | 3.58 | C |
| COV052 | Yes | 1.93 | 0.49 | 3.10 | C |
