# Supplementary material for: Francisella spp. as an overlooked cause of acute undifferentiated febrile illness in Colombia? Unexpected evidence from febrile patients negative for other common and neglected etiologies in Villeta municipality
Source: Trop Med Health. 2026 Jan 3;54:16. doi: 10.1186/s41182-025-00883-6 (PMC12805694; doi:10.1186/s41182-025-00883-6)
Supplement: Supplementary file 2 — Additional file 2. Table 2: General sequencing output parameters and quality control metrics obtained from 16S rRNA barcoding. [file 41182_2025_883_MOESM2_ESM.docx]

**Supplementary Table 2.** General sequencing output parameters and quality control metrics obtained from 16S rRNA barcoding

| **Barcode** | **Pre-merge reads** | **Pre-merge bases (kbp)** | **Post-merge reads** | **Post-merge bases (kbp)** | **Filtered reads** | **Filtered bases (kbp)** | **Reads retained (%)** | **Mean post-filter reads length (bp)** | **Mean post-filter quality (Q)** |
| --- | --- | --- | --- | --- | --- | --- | --- | --- | --- |
| COV003 | 17622 | 17446 | 17622 | 17446 | 10083 | 11777 | 57.2 | 1168 | 16.4 |
| COV017 | 39766 | 40628 | 39766 | 40628 | 21067 | 24584 | 53 | 1166.9 | 16.4 |
| COV019 | 320528 | 358703 | 320528 | 358703 | 207999 | 242882 | 64.9 | 1167.7 | 16.5 |
| COV027 | 387904 | 450244 | 387904 | 450244 | 276150 | 322664 | 71.2 | 1168.4 | 16.5 |
| COV028 | 408982 | 477256 | 408982 | 477256 | 293473 | 342509 | 71.8 | 1167.1 | 16.4 |
| COV031 | 267565 | 311247 | 267565 | 311247 | 181490 | 214445 | 67.8 | 1181.6 | 16.4 |
| COV036 | 455242 | 525928 | 455242 | 525928 | 311793 | 369942 | 68.5 | 1186.5 | 16.4 |
| COV052 | 340813 | 394088 | 340813 | 394088 | 239467 | 282993 | 70.3 | 1181.8 | 16.4 |
