# Supplementary material for: Francisella spp. as an overlooked cause of acute undifferentiated febrile illness in Colombia? Unexpected evidence from febrile patients negative for other common and neglected etiologies in Villeta municipality
Source: Trop Med Health. 2026 Jan 3;54:16. doi: 10.1186/s41182-025-00883-6 (PMC12805694; doi:10.1186/s41182-025-00883-6)
Supplement: Supplementary file 3 — Additional file 3. Table 3: Mapping and taxonomic classification metrics obtained for each processed sample. [file 41182_2025_883_MOESM3_ESM.docx]

**Supplementary Table 3.** Mapping and taxonomic classification metrics obtained for each processed sample.

| **Barcode** | **Filtered reads** | **Mapped reads** | **Classified reads** | **% classified reads** |
| --- | --- | --- | --- | --- |
| COV003 | 10083 | 10052 | 9227 | 98.5 |
| COV017 | 21067 | 20852 | 20633 | 97.9 |
| COV019 | 207999 | 204932 | 202659 | 97.4 |
| COV027 | 276150 | 273651 | 271034 | 98.1 |
| COV028 | 293473 | 292571 | 289362 | 98.6 |
| COV031 | 181490 | 179278 | 177190 | 97.6 |
| COV036 | 311793 | 305821 | 302748 | 97.1 |
| COV052 | 239467 | 238254 | 234270 | 97.8 |
